# Relato de Caso de Doença Coronariana e Vascular Não Aterosclerótica: Em Busca de uma Entidade Clínica Rara

**DOI:** 10.36660/abc.20210722

**Published:** 2022-08-25

**Authors:** Gustavo Sá Mendes, António Epifânio Mesquita, Bruno Rocha, João Abecasis, Sancia Ramos, Marisa Trabulo

**Affiliations:** 1 Serviço Cardiologia Hospital de Santa Cruz Centro Hospitalar Lisboa Ocidental Lisboa Portugal Serviço Cardiologia, Hospital de Santa Cruz, Centro Hospitalar Lisboa Ocidental, Lisboa – Portugal; 2 Serviço Medicina Interna Hospital Santo António dos Capuchos Centro Hospitalar Lisboa Central Lisboa Portugal Serviço Medicina Interna, Hospital Santo António dos Capuchos, Centro Hospitalar Lisboa Central, Lisboa – Portugal; 3 Serviço anatomia Patológica Hospital de Santa Cruz Centro Hospitalar Lisboa Ocidental Lisboa Portugal Serviço anatomia Patológica, Hospital de Santa Cruz, Centro Hospitalar Lisboa Ocidental, Lisboa – Portugal

**Keywords:** Doença Arterial Coronariana, Imunoglobulina G4, Aortite, Imagem Multimodal/métodos, Inflamação, Insuficiência Cardíaca, Disfunção Ventricular Esquerda, Diagnóstico por Imagem/métodos

## Introdução

Aqui relatamos um caso desafiador de uma condição sistêmica rara – doença relacionada à imunoglobulina G4 (IgG4-RD) –que se apresentou com uma rara manifestação cardiovascular. A aortite por IgG4-RD está bem documentada na literatura, mas raramente tem sido relacionada ao envolvimento da árvore arterial coronariana.^1^ Documentamos IgG4-RD com periarterite coronariana difusa, apresentando-se como insuficiência cardíaca aguda neste caso particular. Apesar da gravidade inicial, o trabalho em equipe multidisciplinar foi a chave para o diagnóstico rápido e o início do tratamento personalizado salvavidas, visando inflamação sistêmica e envolvimento de órgãos autoimunes.

### Apresentação do caso

Uma mulher caucasiana de 56 anos deu entrada no pronto-socorro com dor torácica atípica, dispneia, cansaço e ocasionais surtos de dor abdominal durante a semana anterior. Além disso, foram relatadas mialgias proximais intermitentes de membros inferiores, ondas de calor cervicais, xerostomia e xeroftalmia. A paciente estava afebril, normotensa com taquicardia sinusal (113 batimentos/min) e taquipneia. Ao exame físico notava-se galope S3 e sinais de congestão pulmonar (ausência de edema periférico). A história médica pregressa era notável por rinite alérgica, emagrecimento (40 Kg, 151 cm) e uso continuado de tabaco (27 maços-ano). Há cinco anos, a paciente apresentava cãibras persistentes em membros inferiores há 5 meses, com estudo ultrassonográfico Doppler arterial sem alterações, tratada com terapia não esteroidal.

A investigação diagnóstica na emergência revelou níveis ligeiramente elevados de troponina T de alta sensibilidade (73 ng/mL), níveis aumentados de peptídeo natriurético do tipo N-terminal pró-B (NT-proBNP) (3485 pg/mL), bem como uma ligeira elevação da creatina quinase. O eletrocardiograma mostrou extrassístoles ventriculares e alterações inespecíficas da repolarização. A ecocardiografia transtorácica (ETT) foi notável por disfunção sistólica grave do ventrículo esquerdo (VE) (fração de ejeção do VE [FEVE] <30%) com hipocinesia global, padrão de enchimento restritivo e regurgitação valvar aórtica moderada (Vídeo 1).

**Vídeo 1:** >Ecocardiograma transtorácico na avaliação inicial: dilatação grave do VE (219ml/m2) com função do VE deprimida (FEVE 25% PAS; strain longitudinal global -8,1%) devido à hipocinesia difusa e global. Acesse o vídeo pelo link: Link: http://abccardiol.org/supplementary-material/2022/11903/2021-0722_CC_video-1.mp4

**Figura 1:**
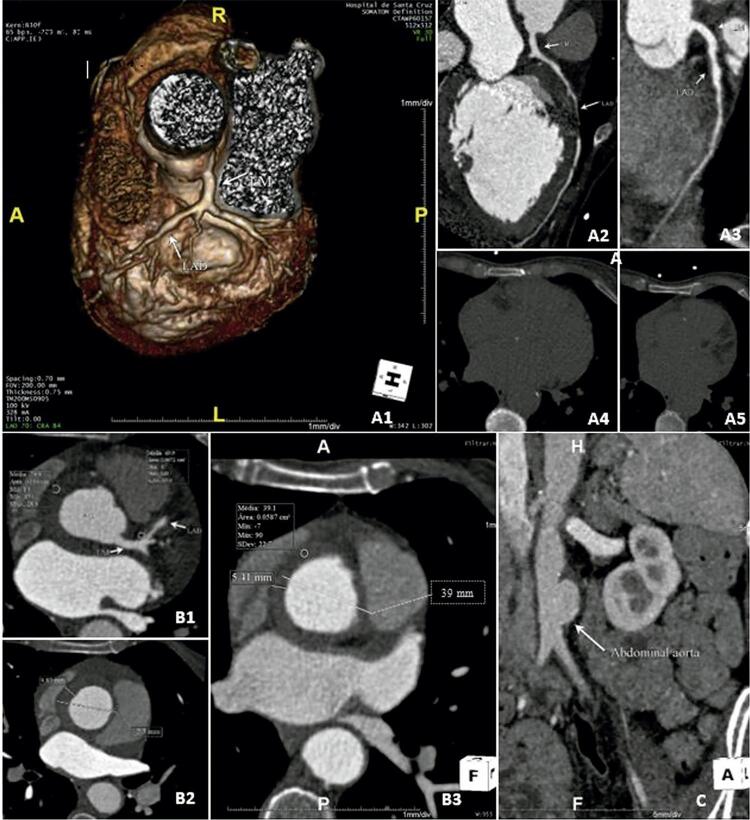
Angio-TC cardíaca Painel A: Infiltração fibrolipídica difusa da artéria coronária, além de aneurisma proximal da descendente anterior esquerda (LAD) (Painéis A1 e A2) e suboclusão da DA do leito distal (painel A3). A densidade de infiltração da parede da artéria coronária é semelhante à densidade da parede da aorta (painel B1). Os painéis A4 e A5 não mostraram cálcio nas artérias coronárias. Painel B: Painel B1 e B2 - avaliação inicial: espessamento homogêneo de 10mm de baixa densidade (~70 unidades Hounsfield [HU]) da parede aórtica, sem aumento de densidade após injeção de contraste. Painel B3 - 15 dias após início do tratamento direcionado: redução do espessamento da parede parietal (10mm a 5mm), do diâmetro máximo da raiz aórtica (43mm a 39mm) e da densidade da parede parietal antes da injeção de contraste (para 40 HU) . Painel C: Angio-TC abdominal mostrou aneurisma de aorta abdominal (47mm) com trombo mural antes do tratamento.

Para excluir doença arterial coronariana e investigar dor abdominal persistente, foi realizada angiotomografia computadorizada (TC) toracoabdominal. Embora não tenha sido observado cálcio na artéria coronária, foi documentada infiltração fibrolipídica difusa da artéria coronária, além de aneurisma proximal da descendente anterior esquerda (DAE) e suboclusão distal da DA. Foi identificada dilatação da aorta ascendente (42 mm) com espessamento concêntrico homogêneo da parede de baixa densidade e aneurisma de aorta abdominal (47 mm) com trombo mural ( Figura 1 ). A angiografia coronária confirmou resultados de Angio TC (doença intermediária difusa com leito distal fino), revascularização não amenizável.

**Figura 2:**
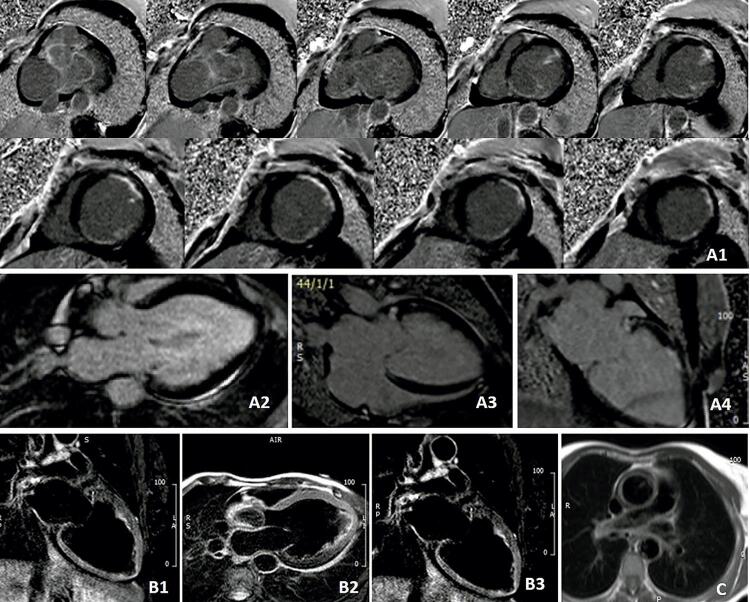
Ressonância magnética cardíaca: A) Sequência escura de realce tardio com gadolínio, mostrando realce tardio difuso não transmural, confirmando cicatriz isquêmica subendocárdica em múltiplos territórios arteriais; B) A sequência T2-W revelou ausência de edema miocárdico (confirmado com mapeamento T2 normal) com edema de parede aórtica, percebido como hipersinal (ponta de seta); C) Sequência T2-STIR suportando o espessamento da parede aórtica.

A ressonância magnética cardíaca (RMC) diagnosticou disfunção do VE com dilatação grave (FEVE 21%; índice de volume diastólico final do VE: 229mL/m2) e realce tardio subendocárdico difuso por gadolínio. As sequências ponderadas em T2 não mostraram edema miocárdico, embora um sinal hiperintenso ao nível da parede da raiz da aorta (Vídeo 2; Figura 2 ).

**Vídeo 2:** Ressonância magnética cardíaca: A) Cine Eixo Curto SSFP; B) Visão de 2 câmaras do SSFP; C) Visão de 4 câmaras do SSFP; D) SSFP Visão do eixo longo. Disfunção sistólica do ventrículo esquerdo com dilatação grave do VE. Acesse o vídeo pelo link: Links: A) Link: http://abccardiol.org/supplementary-material/2022/11903/2021-0722_CC_video-2A.mp4

**Vídeo 2:** Ressonância magnética cardíaca: A) Cine Eixo Curto SSFP; B) Visão de 2 câmaras do SSFP; C) Visão de 4 câmaras do SSFP; D) SSFP Visão do eixo longo. Disfunção sistólica do ventrículo esquerdo com dilatação grave do VE. Acesse o vídeo pelo link: Links: B) Link: Link: http://abccardiol.org/supplementary-material/2022/11903/2021-0722_CC_video-1.mp4

**Vídeo 2:** Ressonância magnética cardíaca: A) Cine Eixo Curto SSFP; B) Visão de 2 câmaras do SSFP; C) Visão de 4 câmaras do SSFP; D) SSFP Visão do eixo longo. Disfunção sistólica do ventrículo esquerdo com dilatação grave do VE. Acesse o vídeo pelo link: Links: C) Link: http://abccardiol.org/supplementary-material/2022/11903/2021-0722_CC_video-2C.mp4

**Vídeo 2:** Ressonância magnética cardíaca: A) Cine Eixo Curto SSFP; B) Visão de 2 câmaras do SSFP; C) Visão de 4 câmaras do SSFP; D) SSFP Visão do eixo longo. Disfunção sistólica do ventrículo esquerdo com dilatação grave do VE. Acesse o vídeo pelo link: Links: D) Link: http://abccardiol.org/supplementary-material/2022/11903/2021-0722_CC_video-2D.mp4

Dada a doença polivascular difusa e a alta suspeita de etiologia não aterosclerótica, foi realizada extensa investigação diagnóstica. Os painéis de investigação de doenças infecciosas e imunológicas (sífilis, citomegalovírus, vírus da hepatite B e C, vírus Epstein Barr, complemento, crioglobulinas, antinucleares, SCL70, Jo1; anticorpos anti-GBM, ECA e lúpus) estavam todos dentro da normalidade, exceto para uma alta velocidade de hemossedimentação (VHS) e hipergamaglobulinemia policlonal. Notavelmente, os níveis séricos de IgG4 aumentaram (1100 mg/L: valor de referência < 291 mg/L).

**Figura 3:**
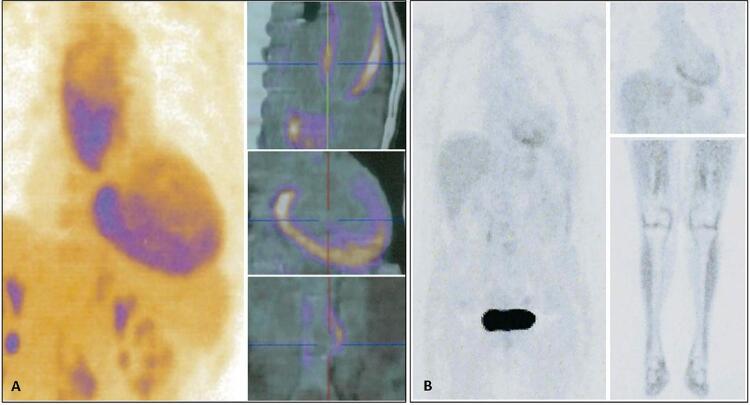
Painel A – PET-CT (Fluorodesoxiglicose) com alta atividade metabólica na aorta ascendente e infrarrenal (*). Ligeira atividade difusa no miocárdio (ponta de seta). Este estudo de imagem também foi notável pelo metabolismo anormal nas carótidas e no eixo arterial dos membros inferiores (possivelmente explicando a sensação de calor no pescoço e as mialgias). Painel B – PET-CT de corpo inteiro (Fluorodesoxiglicose) após um ano de tratamento: resolução completa com atividade metabólica normal no miocárdio, aorta ascendente e infrarrenal, carótidas e eixo dos membros inferiores.

Um estudo de imagem adicional com tomografia por emissão de pósitrons (PET) - TC mostrou intensa atividade do traçador sobre a aorta ascendente proximal e infrarrenal ( Figura 3A ).

**Figura 4:**
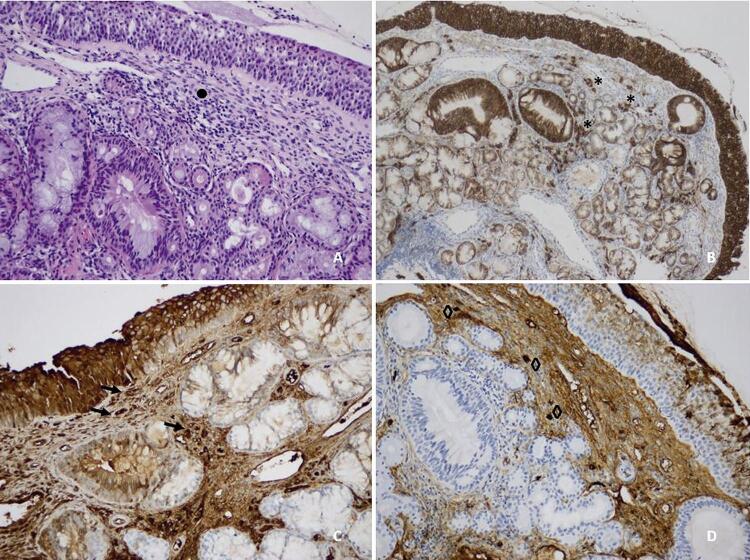
Biópsia da mucosa nasal: Painel A- HE x20: infiltrado linfoplasmocitário difuso e denso (•); Painel B- CD138 x20: população plasmocítica (*); Painel C-IgG x40: infiltrado de células IgG (ponta de seta); Painel D- IgG4 x40, revelando a supobupolação de células IgG4 (◊). A biópsia foi em sua maioria pouco informativa e não apresentou um denso infiltrado linfocitário, destacando o envolvimento heterogêneo de órgãos característico de IgG4-RD. Apesar disso, houve um leve aumento de plasmócitos da lâmina própria (CD138+); muitos foram positivos para IgG e mesmo com um infiltrado inflamatório tão escasso, foi calculada uma razão IgG4+/IgG+ de 23,1%. Isso se encaixa nos critérios EULAR: proporção IgG4+/IgG+ de 0-40% e um número indeterminado de células IgG4+/HPF.

Em relação à história prévia de rinite alérgica, foi realizada biópsia da mucosa nasal encontrando denso infiltrado linfocitário e discreto aumento de plasmócitos da lâmina própria (CD138+), muitos deles positivos para IgG4+ (razão IgG4+/IgG+ de 0-40% e um número indeterminado de células IgG4+/HPF) ( Figura 4 ). Diante de todo o exposto, suspeitou-se do diagnóstico de IgG4-RD quando consideramos os critérios EULAR ( Tabela 1 ).^1^

**Tabela 1:** Critérios EULAR para doença relacionada a IgG4

Critério de entrada	
Envolvimento clínico ou radiológico característico* de um órgão típico (por exemplo, pâncreas, glândulas salivares, ductos biliares, órbitas, rim, pulmão, aorta, retroperitônio, paquimeninges ou glândula tireoide). OU Evidência patológica de processo inflamatório acompanhado por infiltrado linfoplasmocitário de etiologia incerta em um desses mesmos órgãos.	
Critério de inclusão	Pontos
**Histopatologia**	
Biópsia não informativa	0
Infiltrado linfocítico denso	+4
Infiltrado linfocítico denso e flebite obliterativa	+6
Infiltrado linfocítico denso e fibrose estoriforme com ou sem flebite obliterativa	+13
***Imunocoloração***	
- IgG4+: a proporção de IgG+ é de 0–40% ou indeterminada, e o número de células IgG4+/HPF é de 0–9.	0
- IgG4+: a proporção de IgG+ é ≥41%, e o número de células IgG4+/HPF é 0–9 ou indeterminado;	+7
- IgG4+: a proporção de IgG+ é de 0–40% e o número de células IgG4+/HPF é ≥10 ou indeterminado.**	+7
- IgG4+: a proporção de IgG+ é de 41 a 70% e o número de células IgG4+/HPF é ≥10	+14
- IgG4+: a proporção de IgG+ é ≥71% e o número de células IgG4+/HPF é 10–50.	+14
- IgG4+: a proporção de IgG+ é ≥71%, e o número de células IgG4+/HPF é ≥51.	+16
**Concentração sérica de IgG4**	
Normal ou não verificado	0
> Normal, mas <2x limite superior do normal	+4
2–5x limite superior do normal	+6
>5x limite superior do normal	+11
**Glândulas lacrimais, parótidas, sublinguais e submandibulares bilaterais**	
Nenhum conjunto de glândulas envolvidas	0
Um conjunto de glândulas envolvidas	+6
Dois ou mais conjuntos de glândulas envolvidas	+14
**Peito**	
Não verificado, ou nenhum dos itens listados está presente	0
Espessamento peribroncovascular e septal	+4
Tecido mole semelhante a uma faixa paravertebral no tórax	+10
**Pâncreas e árvore biliar**	
Não verificado ou nenhum dos itens listados está presente	0
Aumento difuso do pâncreas (perda de lobulações)	+8
Aumento difuso do pâncreas e borda em forma de cápsula com realce diminuído	+11
Pâncreas (qualquer uma das opções acima) e envolvimento da árvore biliar	+19
**Rim**	
Não verificado ou nenhum dos itens listados está presente	0
Hipocomplementemia	+6
Espessamento da pelve renal/tecido mole	+8
Áreas de baixa densidade do córtex renal bilateral	+1
**Retroperitônio**	
Não verificado, ou nenhum dos itens listados está presente	0
Espessamento difuso da parede da aorta abdominal	+4
Tecido mole circunferencial ou anterolateral ao redor da aorta infrarrenal ou artérias ilíacas	+8

Se os critérios de entrada forem atendidos, o caso atende aos critérios de classificação para IgG4-RD e o total de pontos dos critérios de inclusão é ≥20. É importante notar que apenas o item de maior peso em cada domínio é pontuado. * Refere-se ao aumento ou massa semelhante a tumor em um órgão afetado, exceto no seguinte: 1) os ductos biliares, onde o estreitamento tende a ocorrer; 2) a aorta, onde é típico o espessamento da parede ou dilatação aneurismática; e 3) os pulmões, onde é comum o espessamento dos feixes broncovasculares. ** “Indeterminado” refere-se a uma situação em que o patologista não pode quantificar claramente o número de células coradas positivamente dentro de um infiltrado, mas ainda pode verificar se o número de células é de pelo menos 10/campo de alta potência (HPF). Por muitas razões, na maioria das vezes sobre a qualidade da imunocoloração.

Dessa forma, o paciente foi iniciado com corticoterapia em altas doses (1000 mg de metilprednisolona nos primeiros 3 dias, seguido de 1mg/kg/dia por 2 meses com redução gradual depois) somada a 6 ciclos de infusão de ciclofosfamida e administração subcutânea de metotrexato. Foram iniciados medicamentos modificadores da doença da insuficiência cardíaca e o paciente foi encaminhado ao nosso centro de reabilitação cardíaca. A angio-TC repetida na alta (15 dias após o início do tratamento direcionado) mostrou uma redução significativa no espessamento da parede aórtica (10 a 5 mm) ( Figura 1B ).

No seguimento de um ano, houve melhora da capacidade funcional, avaliada pela classe NYHA e valor de pico V02 (13,8 a 19,9 ml/kg/min), redução nos níveis de NT-proBNP (5260 a 2052 pg/mL) e sinais de remodelação cardíaca reversa (nomeadamente melhoria da FEVE de 30 para 40%). Além disso, houve declínio progressivo dos marcadores de doença inflamatória para valores normais (IgG4 1100 a 83 mg/dl, VHS 42 a 10 mm/h) e resolução completa da atividade metabólica anormal na reavaliação do PET CT ( Figura 3B ).

## Discussão

A IgG4-RD é uma condição fibroinflamatória imunomediada de múltiplos órgãos caracterizada por infiltração tecidual difusa de plasmócitos IgG4 positivos, fibrose estoriforme, flebite obliterativa e aumento de IgG sérica4.^1 , 2^ Este caso descreve uma apresentação anedótica e complexa com aortite e arterite coronária concomitante. A arterite crônica é uma apresentação típica de IgG4-RD envolvendo as grandes e, menos frequentemente, as artérias de médio calibre.^3 , 4^ A doença arterial coronariana é raramente relatada e, até onde sabemos, este é o único relato de caso em que a insuficiência cardíaca aguda foi o gatilho para a investigação inicial.^5 , 6^

Os autores afirmam que a origem não aterosclerótica, neste caso, foi suspeitada pela ausência de cálcio coronariano (escore de cálcio 0) – demonstrando o alto valor preditivo negativo para doença calcificada/aterosclerótica clássica; a presença de doença difusa na coronariografia com apenas lesões intermediárias e suboclusão muito distal da DA não explica as alterações cinéticas difusas no ecocardiograma e na RMC. Além disso, contra uma origem aterosclerótica, há LGE subendocárdico difuso na RMC em vez de segmentar. Além disso, a história clínica florida deste caso é mais bem explicada por uma doença multissistêmica, apesar de uma etiologia tipicamente não aterosclerótica.

A imagem multimodal aliada à assinatura inflamatória foi essencial para esse desafio diagnóstico e terapêutico. Técnicas de imagem cardiovascular, como ETT, angio-TC, PET-CT e RMC, foram usadas com sucesso para detecção de doenças, avaliação de sintomas e monitoramento. Enquanto a VHS e a hipergamaglobulinemia policlonal levantaram a suspeita de uma possível vasculite, o espessamento da parede aórtica e a doença arterial coronariana não aterosclerótica foram primordiais para orientar a investigação para uma etiologia que não fosse a aterosclerose clássica. PET-CT confirmou a inflamação periarterial e coronária ativa. Além disso, forneceu pistas para correlação clínica, ou seja, explosões de dor abdominal, mialgia proximal dos membros inferiores e ondas de calor cervicais (como observado pela atividade metabólica/inflamatória difusa de todo o corpo). De acordo com um grande estudo retrospectivo, a imagem PET-CT pode ser a única modalidade de imagem útil para avaliar a resposta ao tratamento durante o seguimento,^7 , 8^ no entanto, também repetimos a angio-TC demonstrando uma melhora significativa da aortite. Devido à sua capacidade de realizar avaliação funcional e caracterização tecidual, a RMC permite a avaliação simultânea da atividade da doença e repercussões específicas na função do VE na presença de arterite coronariana.

Uma equipe multidisciplinar (Cardiologia, Reumatologia, Medicina Nuclear e Patologia) foi de extrema importância na investigação da multiplicidade de envolvimento de órgãos e chave para o diagnóstico de IgG4-RD. Após descartar o diagnóstico mais frequente, a equipe multidisciplinar, com base em todos os achados clínicos e laboratoriais, decide assumir a doença relacionada à IgG4. Embora a biópsia não tenha sido totalmente patogênica, ela foi realizada em local não ativamente afetado neste momento (mas na história médica prévia); optamos por não realizar biópsia aórtica ou miocárdica na fase aguda e instável com riscos potencialmente maiores. Embora não tenhamos um diagnóstico confirmador de envolvimento glandular, a reumatologia, essencial neste caso, considera bastante típico o envolvimento de glândulas salivares e xeroftalmia.

O bloqueio neuro-hormonal com agentes modificadores da doença é essencial para melhorar a sobrevida em pacientes com Insuficiência Cardíaca e reduzir a FEVE. De acordo com o Consenso Internacional sobre o tratamento de IgG4-RD, os glicocorticoides são os agentes de primeira linha para indução da remissão,^9^ mesmo nos estágios fibróticos avançados.^10^ As lesões cardiovasculares relacionadas à IgG4 geralmente requerem doses mais altas de corticosteroides,^11 , 12^ muitas vezes melhorando as lesões inflamatórias na TC ou PET.^7^ Este caso foi ainda mais complicado pelo efeito mineralocorticoide derivado dos corticosteroides, que pode facilitar a descompensação da Insuficiência Cardíaca. Embora dados observacionais possam apoiar essa abordagem, o tratamento inicial com combinação de imunossupressores permanece controverso.^13^ A ciclofosfamida demonstrou ter bons resultados a longo prazo e menores taxas de recaída.^14^ Da mesma maneira, rituximabe também foi sugerido para ter efeitos benéficos no IGG4-RD, mas reduziu severamente os sintomas de FEVE e Insuficiência Cardíaca e tuberculose latente, contraindicando seu uso em nosso caso. Diferentemente do caso publicado anteriormente envolvendo artérias coronárias,^5^ na discussão multidisciplinar, consideramos que a aspirina não teve nenhum papel nesse tipo de acometimento arterial e aumentou o risco de sangramento devido ao tratamento contínuo com esteroides.

Relatamos um caso desafiador de IgG4-RD apresentando insuficiência cardíaca aguda consequente a arterite coronariana e aortite, com tratamento conservador bem-sucedido, em vez de cirurgia de revascularização miocárdica invasiva como estratégia inicial.^6^ Além de ser notável por sua rara apresentação, este caso destaca o papel da multimodalidade de imagem e da investigação multidisciplinar como peças-chave no estabelecimento correto de um diagnóstico e na facilitação de um plano de tratamento personalizado.
