# Supplementary material for: Relato de Caso de Doença Coronariana e Vascular Não Aterosclerótica: Em Busca de uma Entidade Clínica Rara
Source: Arq Bras Cardiol. 2022 Aug 25;119(3):488–95. [Article in Portuguese] doi: 10.36660/abc.20210722 (PMC9438549; doi:10.36660/abc.20210722)
Supplement: Translation

# Translation: Non-Atherosclerotic Coronary and Vascular Disease Case Report: Searching for a Rare Clinical Entity

**Authors:** Gustavo Sá Mendes, António Epifânio Mesquita, Bruno Rocha, João Abecasis, Sancia Ramos, Marisa Trabulo

**Affiliations:** 1 Portugal Serviço Cardiologia, Hospital de Santa Cruz, Centro Hospitalar Lisboa Ocidental, Lisboa – Portugal; 2 Portugal Serviço Medicina Interna, Hospital Santo António dos Capuchos, Centro Hospitalar Lisboa Central, Lisboa – Portugal; 3 Portugal Serviço anatomia Patológica, Hospital de Santa Cruz, Centro Hospitalar Lisboa Ocidental, Lisboa – Portugal

**Keywords:** Coronary Artery Disease, Immunoglobulin G4-Related Disease, Aortitis, Multimodal Imaging/methods, Inflammation, Heart Failure, Ventricular Dysfunction, Left, Diagnostic,Imaging/methods

## Introduction

Here we report a challenging case of a rare systemic condition – immunoglobulin G4-related disease (IgG4-RD) – that presented with a rare cardiovascular manifestation. Aortitis due to IgG4-RD is well-documented in the literature, but rarely has it been related to the involvement of the coronary arterial tree.^1^ We documented IgG4-RD with diffuse coronary periarteritis, presenting as acute heart failure in this particular case. Despite the initial severity, multidisciplinary teamwork was the key to expeditious diagnosis and initiating tailored life-saving treatment targeting systemic inflammation and autoimmune organ involvement.

## Case presentation

A 56-year-old Caucasian woman presented to the emergency department with atypical chest pain, dyspnea, tiredness, and occasional abdominal pain bursts during the previous week. In addition, intermittent proximal lower limb myalgia, cervical hot flashes, xerostomy and xerophthalmia were reported. The patient was afebrile, normotensive with sinus tachycardia (113 beats/min) and tachypnea. The physical examination was remarkable for S3 gallop and signs of pulmonary congestion (no peripheral edema). Past medical history was notable for allergic rhinitis, emaciation (40 Kg, 151 cm) and continued tobacco use (27 pack-year). Five years before, the patient had persistent lower limb cramps for 5 months, with an unremarkable arterial Doppler ultrasound study, treated with non-steroidal therapy.

Diagnostic workup at the emergency revealed slightly elevated high sensitivity troponin T levels (73 ng/mL), increased levels of N-terminal pro–B-type natriuretic peptide (NT- proBNP) (3485 pg/mL) as well as a slight elevation of creatine kinase. Electrocardiography showed ventricular extrasystoles and non-specific repolarization abnormalities. Transthoracic echocardiography (TTE) was remarkable for severe left ventricular (LV) systolic dysfunction (LV ejection fraction [LVEF] <30%) with global hypokinesia, restrictive filling pattern and moderate aortic valve regurgitation (Video 1).

**Video 1:** Transthoracic echocardiogram at initial evaluation: severe LV dilation (219ml/m2) with depressed LV function (LVEF 25% SBP; Global longitudinal strain -8.1%) due to diffuse and global hypokinesia. Acesse o vídeo pelo link: Link: http://abccardiol.org/supplementary-material/2022/11903/2021-0722_CC_video-1.mp4

In order to exclude coronary artery disease and investigate persistent abdominal pain, a thoracoabdominal computed tomographic (CT) angiography was performed. While no coronary artery calcium was observed, diffuse coronary artery fibrolipidic infiltration was documented, in addition to proximal left anterior descendent (LAD) aneurysm and distal LAD suboclusion. Dilatation of the ascending aorta (42 mm) with concentric homogeneous low-density wall thickening and an abdominal aorta aneurysm (47mm) with mural thrombus was identified ( Figure 1 ). Coronary angiography confirmed Angio CT (diffuse intermediate disease with thin distal bed) results, not amenable revascularization.

**Figure 1:** Cardiac angio-CT Panel A: Diffuse coronary artery fibrolipidic infiltration, in addition to proximal left anterior descendent (LAD) aneurysm (Panel A1 and A2) and distal bed LAD suboclusion (panel A3). Coronary artery wall infiltration density is similar to aortic wall density (panel B1). Panel A4 and A5 showed no coronary artery calcium. Panel B: Panel B1 and B2 - initial evaluation: 10mm homogeneous low-density (~70 Hounsfield unit [HU]) aortic wall thickening, with no increased density after contrast dye injection. Panel B3 - 15 days after starting targeted treatment: reduction in thickening of the parietal wall (10mm to 5 mm), in aortic root maximum diameter (43mm to 39mm) and the density of the parietal wall before the contrast injection (to 40 HU). Panel C: Abdominal angio-CT showed abdominal aorta aneurysm (47mm) with mural thrombus before treatment.

Cardiac magnetic resonance (CMR) diagnosed LV dysfunction with severe dilatation (LVEF 21%; LV end-diastolic volume index: 229mL/m^2^) and diffuse subendocardial late gadolinium enhancement. T2-weighed sequences showed no myocardial edema, albeit a hyperintense signal at the aortic root wall level (Video 2; Figure 2).

**Video 2:** Cardiac magnetic resonance: A) SSFP Short Axis Cine; B) SSFP 2 chamber view; C) SSFP 4 chamber view; D) SSFP Long axis view. Left ventricular systolic dysfunction with severe LV dilatation. Links: A) Link: http://abccardiol.org/supplementary-material/2022/11903/2021-0722_CC_video-2A.mp4

**Video 2:** Cardiac magnetic resonance: A) SSFP Short Axis Cine; B) SSFP 2 chamber view; C) SSFP 4 chamber view; D) SSFP Long axis view. Left ventricular systolic dysfunction with severe LV dilatation. Links: B) Link: Link: http://abccardiol.org/supplementary-material/2022/11903/2021-0722_CC_video-1.mp4

**Video 2:** Cardiac magnetic resonance: A) SSFP Short Axis Cine; B) SSFP 2 chamber view; C) SSFP 4 chamber view; D) SSFP Long axis view. Left ventricular systolic dysfunction with severe LV dilatation. Links: C) Link: http://abccardiol.org/supplementary-material/2022/11903/2021-0722_CC_video-2C.mp4

**Video 2:** Cardiac magnetic resonance: A) SSFP Short Axis Cine; B) SSFP 2 chamber view; C) SSFP 4 chamber view; D) SSFP Long axis view. Left ventricular systolic dysfunction with severe LV dilatation. Links: D) Link: http://abccardiol.org/supplementary-material/2022/11903/2021-0722_CC_video-2D.mp4

**Figure 2:** Cardiac magnetic resonance: A) Late gadolinium enhancement dark blood sequence, showing diffuse non-transmural late enhancement, confirming subendocardial ischemic scar across multiple artery territories; B) T2-W sequence revealed the absence of myocardial edema (confirmed with normal T2 mapping) with aortic wall edema, appreciated as a hyperintense signal (arrowhead); C) T2-STIR sequence supporting the aortic wall thickening.

Given the diffuse polivascular disease and as a non-atherosclerotic etiology was highly suspected, an extensive diagnostic workup was performed. Infectious and immunologic disease investigation panels (syphilis, cytomegalovirus, hepatitis B and C virus, Epstein Barr virus, complement, cryoglobulins, anti-nuclear, SCL70, Jo1; anti- GBM, ECA and lupus antibodies) were all within normal range, except for a high erythrocyte sedimentation rate (ESR) and polyclonal hypergammaglobulinemia. Notably, serum levels of IgG4 were increased (1100 mg/L: reference value < 291 mg/L).

An additional imaging study with Positron Emission Tomography (PET) - CT depicted intense tracer activity over the proximal ascending and infra-renal aorta ( Figure 3A ).

**Figure 3:** Panel A – PET-CT (Fluorodeoxyglucose) with high metabolic activity across ascending and infrarrenal aorta (*). Slight diffuse activity in the myocardium (arrowhead). This imaging study was also notable for the abnormal metabolism across the carotids and lower limb arterial axis (possibly explaining the heat neck sensation and myalgias). Panel B – Whole body PET-CT (Fluorodeoxyglucose) after one-year treatment: complete resolution with normal metabolic activity across myocardium, ascending and infra-renal aorta, carotids, and lower limb axis.

Regarding the previous history of allergic rhinitis, a nasal mucosal biopsy was performed: it found a dense lymphocytic infiltrate and a slight increase in lamina propria plasma cells (CD138+), many of these positive for IgG4+ (an IgG4+/IgG+ ratio of 0-40% and an indeterminate number of IgG4+ cells/HPF) ( Figure 4 ). Given all the above, the diagnosis of IgG4-RD was suspected when we considered the EULAR criteria ( Table 1 ).^1^

**Figure 4:** Nasal mucosa biopsy: Panel A- HE x20: diffuse and dense lymphoplasmacytic infiltrate (•); Panel B- CD138 x20: plasmacytic population (*); Panel C- IgG x40: infiltrate of IgG cells (arrowhead); Panel D- IgG4 x40, revealing the supobupolation of IgG4 cells (◊). The biopsy was mostly uninformative and lacked a dense lymphocytic infiltrate, highlighting the heterogeneous organ involvement characteristic of IgG4-RD. Nevertheless, there was a slight increase in lamina propria plasma cells (CD138+); many were positive for IgG and even with such a sparse inflammatory infiltrate, an IgG4+/IgG+ ratio of 23.1% was calculated. This fits the criteria EULAR: IgG4+/IgG+ ratio of 0-40% and an indeterminate number of IgG4+ cells/HPF.

**Table 1:** EULAR criteria for IgG4- Related disease

Entry Criteria	
Characteristic* clinical or radiologic involvement of a typical organ (e.g., pancreas, salivary glands, bile ducts, orbits, kidney, lung, aorta, retroperitoneum, pachymeninges, or thyroid gland OR Pathologic evidence of an inflammatory process accompanied by a lymphoplasmacytic infiltrate of uncertain etiology in one of these same organs.	
Inclusion Criteria	Points
**Histopathology**	
Uninformative biopsy	0
Dense lymphocytic infiltrate	+4
Dense lymphocytic infiltrate and obliterative phlebitis	+6
Dense lymphocytic infiltrate and storiform fibrosis with or without obliterative phlebitis	+13
***Immunostaining***	
- IgG4+: IgG+ ratio is 0–40% or indeterminate, and the number of IgG4+ cells/HPF is 0–9.	0
- IgG4+: IgG+ ratio is ≥41%, and the number of IgG4+ cells/HPF is 0–9 or indeterminate;	+7
- IgG4+: IgG+ ratio is 0–40%, and the number of IgG4+ cells/HPF is ≥10 or indeterminate.**	+7
- IgG4+: IgG+ ratio is 41–70%, and the number of IgG4+ cells/HPF is ≥10	+14
- IgG4+: IgG+ ratio is ≥71%, and the number of IgG4+ cells/ HPF is 10–50.	+14
- IgG4+: IgG+ ratio is ≥71%, and the number of IgG4+cells/ HPF is ≥51.	+16
**Serum IgG4 concentration**	
Normal or not checked	0
> Normal but <2x upper limit of normal	+4
2–5x upper limit of normal	+6
>5x upper limit of normal	+11
**Bilateral lacrimal, parotid, sublingual, and submandibular glands**	
No set of glands involved	0
One set of glands involved	+6
Two or more sets of glands involved	+14
**Chest**	
Not checked, or neither of the items listed is present	0
Peribronchovascular and septal thickening	+4
Paravertebral band-like soft tissue in the thorax	+10
**Pancreas and biliary tree**	
Not checked, or none of the items listed is present	0
Diffuse pancreas enlargement (loss of lobulations)	+8
Diffuse pancreas enlargement and capsule-like rim with decreased enhancement	+11
Pancreas (either of the above) and biliary tree involvement	+19
**Kidney**	
Not checked, or none of the items listed is present	0
Hypocomplementemia	+6
Renal pelvis thickening/soft tissue	+8
Bilateral renal cortex low-density areas	+1
**Retroperitoneum**	
Not checked, or neither of the items listed is present	0
Diffuse thickening of the abdominal aortic wall	+4
Circumferential or anterolateral soft tissue around the infrarenal aorta or iliac arteries	+8

If the entry criteria are met, the case meets the classification criteria for IgG4-RD, and the total points of inclusion criteria are ≥20. Of note, only the highest-weighted item in each domain is scored.^*^Refers to enlargement or tumor-like mass in an affected organ except in the following: 1) the bile ducts, where narrowing tends to occur; 2) the aorta, where wall thickening or aneurysmal dilatation is typical; and 3) the lungs, where thickening of the bronchovascular bundles is common.^**^“Indeterminate” refers to a situation in which the pathologist cannot clearly quantify the number of positively staining cells within an infiltrate yet can still ascertain that the number of cells is at least 10/high-power field (HPF). For many reasons, most often about the quality of the immunostaining, pathologists are sometimes unable to count the number of IgG4+ plasma cells with precision, yet, even so, can be confident in grouping cases into the appropriate immunostaining result category.

Accordingly, the patient was started on high-dose corticosteroid therapy (1000 mg of methylprednisolone in the first 3 days, followed by 1mg/kg/day for 2 months with slow tapering afterward) added to 6 cycles of cyclophosphamide infusion and methotrexate subcutaneous administration. Heart failure disease-modifying drugs were initiated, and the patient was referred to our cardiac rehabilitation center. Repeated angio-CT at discharge (15 days after the start of targeted treatment) showed a significant reduction in aortic wall thickening (10 to 5 mm) ( Figure 1B ).

At one year follow-up, there was an improvement in functional capacity, as assessed by NYHA class and peak V02 value (13.8 to 19.9 ml/kg/min), a reduction in NT-proBNP levels (5260 to 2052 pg/mL) and signs of reverse cardiac remodeling (namely LVEF improvement from 30 to 40%). Moreover, there was a progressive decline in inflammatory disease markers towards normal values (IgG4 1100 to 83 mg/dl, ESR 42 to 10 mm/h) and complete resolution of abnormal metabolic activity at PET CT reassessment ( Figure 3B ).

## Discussion

IgG4-RD is a multi-organ immune-mediated fibroinflammatory condition characterized by diffuse tissue infiltration of IgG4-positive plasma cells, storiform fibrosis, obliterative phlebitis, and increased serum IgG4.^1 , 2^ This case describes an anecdotally reported and complex presentation with aortitis and concomitant coronary arteritis. Chronic arteritis is a typical presentation of IgG4-RD involving the large and, less frequently, medium-sized arteries.^3 , 4^ Coronary artery disease is seldom reported, and to the best of our knowledge, this is the only case report in which acute heart failure was the trigger for the initial investigation.^5 , 6^

The authors state that the non-atherosclerotic origin, in this case, was suspected due to the absence of coronary calcium (calcium score 0)- demonstrating the high negative predictive value for classic calcified/atherosclerotic disease; the presence of diffuse disease on coronary angiography with only intermediate lesions and very distal LAD suboclusion does not explain the diffuse kinetic changes on echocardiography and CMR. Also, against an atherosclerotic origin, there is diffuse subendocardial LGE at CMR rather than segmental. Furthermore, the flowery clinical history of this case is better explained by a multisystemic disease, despite a typically non-atherosclerotic etiology.

Multimodality imaging coupled with the inflammatory signature was essential for this diagnostic and therapeutic challenge. Cardiovascular imaging techniques, namely TTE, angio-CT, PET-CT and CMR, were successfully used for disease detection, symptom assessment, and monitoring. While ESR and polyclonal hypergammaglobulinemia raised the suspicion of a possible vasculitis, the aortic wall thickening and non-atherosclerotic coronary artery disease were paramount in guiding the investigation toward an etiology different than classical atherosclerosis. PET-CT confirmed the active periarterial and coronary artery inflammation. Moreover, it provided clues for clinical correlation, namely abdominal pain bursts, proximal lower limb myalgia and cervical hot flashes (as noted by diffuse whole body enhanced metabolic/inflammatory activity). According to a large retrospective study, PET-CT imaging may be the only imaging modality useful for assessing treatment response during follow-up;^7 , 8^ nonetheless, we also repeated Angio- CT demonstrating a significant improvement of aortitis. Due to its capability to perform functional evaluation and tissue characterization, CMR allows simultaneous assessment of disease activity and specific repercussions on LV function when coronary arteritis is present.

A multidisciplinary team (Cardiology, Rheumatology, Nuclear Medicine and Pathology) was of utmost importance in investigating the multitude of organ involvement and key to achieving the diagnosis of IgG4-RD. After ruling out the most frequent diagnosis, the multidisciplinary team, based on all clinical and laboratory findings, decides to assume the IgG4 - related disease. Although the biopsy was not fully pathogenic, it was performed in a site not actively affected at this moment (but in the previous medical history); we chose not to perform an aortic or myocardial biopsy in the acute and unstable phase, potentially greater risks. Although we did not have a confirming diagnosis of gland involvement, the rheumatology, essential in this case, considers the involvement of salivary glands and xerophthalmia to be quite typical. Even though the criteria might not be fulfilled, the fact that the patient presented a good response to the IgG4-RD targeted therapy corroborates this hypothesis.

Neuro-hormonal blockade with disease-modifying agents is essential to improve survival in patients with Heart Failure and reduce LVEF. According to the International Consensus on the treatment of IgG4-RD, glucocorticoids are the first-line agents for induction of remission,^9^ even in the advanced fibrotic stages.^10^ IgG4-related cardiovascular lesions usually require higher doses of corticosteroids,^11 , 12^ often improving the inflammatory lesions on CT or PET imaging.^7^ This case was further complicated by the mineralocorticoid-like effect derived from corticosteroids, which can facilitate Heart Failure decompensation. Although observational data may support this approach, initial treatment with combination immunosuppressors remains controversial.^13^ Cyclophosphamide has been shown to have good long-term outcomes and lower relapse rates.^14^ Likewise, rituximab has also been suggested to have beneficial effects in IGG4-RD, yet severely reduced LVEF and Heart Failure symptoms and latent tuberculosis, contra-indicated its use in our case. Unlike the previously published case involving coronary arteries,^5^ in the multidisciplinary discussion, we consider that aspirin had no role in this type of artery involvement and increased the bleeding risk due to ongoing steroid treatment.

We reported a challenging case of IgG4-RD presenting with acute heart failure consequent to coronary arteritis and aortitis, with successful conservative management, rather than invasive coronary bypass graft surgery as an initial strategy.^6^ Besides being remarkable for its rare presentation, this case highlights the role of multimodality imaging and multidisciplinary workup as key players in correctly establishing a diagnosis and facilitating a tailored treatment plan.
